# Influence of different functionalized CdTe quantum dots on the accumulation of metals, developmental toxicity and respiration in different development stages of the zebrafish (*Danio rerio*)

**DOI:** 10.3389/ftox.2023.1176172

**Published:** 2023-05-02

**Authors:** Suanne Bosch, Tarryn Lee Botha, Victor Wepener

**Affiliations:** ^1^ Water Research Group, School of Biological Sciences, North-West University, Potchefstroom, South Africa; ^2^ Department of Zoology, University of Johannesburg, Johannesburg, South Africa

**Keywords:** metal uptake, oxygen consumption, carboxylate (COOH), ammonia (NH_3_), polyethylene glycol (PEG)

## Abstract

**Introduction:** The bioaccumulation and differential effects of cadmium tellurium quantum dot (CdTe QDs) nanomaterials with different functional groups are poorly understood in aquatic organisms. This study aimed to investigate the metal uptake, developmental effects, and respiratory effects of CdTe QDs with different functional groups (COOH, NH3, and PEG) on zebrafish embryos.

**Methods:** Zebrafish embryos were exposed to carboxylate (COOH), ammonia (NH3), and polyethylene glycol (PEG) functionalized CdTe QDs at nominal concentrations of 0.5, 2, 4, 6, and 20 mg QDs/L. The materials were characterized in E3 exposure media and the metal uptake, developmental effects, and respiratory effects of zebrafish embryos were recorded.

**Results:** The total Cd or Te concentrations in the larvae could not be explained by the metal concentrations or dissolution of the materials in the exposure media. The metal uptake in the larvae was not dose-dependent, except for the QD-PEG treatment. The QD-NH3 treatment caused respiration inhibition at the highest exposure concentration and hatching delays and severe malformations at low concentrations. The toxicities observed at low concentrations were attributed to particles crossing the pores in the chorion, and toxicities at higher concentrations were linked to the aggregation of particle agglomerates to the surface of the chorion impairing respiration. Developmental defects were recorded following exposure to all three functional groups, but the QD-NH3 group had the most severe response. The LC50 values for embryo development of QD-COOH and QD-PEG groups were higher than 20 mg/L, and the LC50 of the QD-NH3 group was 20 mg/L.

**Discussion:** The results of this study suggest that CdTe QDs with different functional groups have differential effects on zebrafish embryos. The QD-NH3 treatment caused the most severe effects, including respiration inhibition and developmental defects. These findings provide valuable information for understanding the effects of CdTe QDs on aquatic organisms and highlight the need for further investigation.

## 1 Introduction

Quantum dots (QDs) are fluorescent nanocrystals with a semiconductor metal core, which is usually comprised of CdSe or CdTe (King-Heiden et al., 2009; Zheng et al., 2021). Due to their small size and unique composition, they absorb and emit light at different wavelengths making them ideal as contrasting agents for bio-imaging applications (Michalet et al., 2005). Besides cellular imaging, QDs can be used for a wide range of other applications, such as fixed tissue analysis, spectral encoding of microparticles, and quantitative analysis of ions (Åkerman et al., 2002; Jason et al., 2009). Even though QDs have many advantageous properties they are essentially inorganic semiconductor materials that can be toxic to living organisms (Jason et al., 2009). Many studies report no or little toxicity to living organisms (Jaiswal et al., 2003; Larson et al., 2003; Chen and Gerion, 2004; Voura et al., 2004; Zhang et al., 2013), however, some studies have seen cytotoxicity and other toxic outcomes from exposure to QDs leading to the development of surface coatings and functionalization to minimize toxicity (Derfus et al., 2004; Lovrić et al., 2005; Jason et al., 2009; Zhang et al., 2012b; Hsu et al., 2012). Surface functionalization with organic molecules increases their dispensability in water, making them more biologically compatible and prevents metal core leaching (Medintz et al., 2005; Michalet et al., 2005; Ron, 2006; King-Heiden et al., 2009).

Given their wide range of applications, the substantial growth in production volumes of QDs is predicted and thus entry of QDs and their by-products into waterways is likely (Singh et al., 2020). Even though the exact concentration of QDs in the environment is unknown, this potentially poses risks to aquatic biota. The coatings that make them unique and useful could also be their weakness. As they end up in the environment the coating could become compromised and reveal a possibly toxic core or cause the dissolution of metals from the core (Ron, 2006; Rocha et al., 2017). As QDs degrade they could also react in undesirable ways *in vivo*. It is said that the estimated environmental residence times for QDs range from months to years (Ron, 2006). From this, it is evident that their physicochemical properties and surface functionalization are fundamental in understanding their potential toxicity.

In the aquatic environment processes such as agglomeration patterns, settling, oxidation, interaction with organic material and biotransformation can influence the fate, behaviour and uptake of any nanomaterial (Rocha et al., 2017; Mishra et al., 2021). These aspects are determined by the physicochemical properties of the environment, particle size, shape, coating, and functionalization. Studies have found that particle size and functionalization determine the interaction with biological membranes (such as cell membranes as well as the chorion of an embryo) and biodistribution in the organism (Paatero et al., 2017). Not many studies have looked at the effect of QDs with the same core size and composition but different functionalization on their toxicity, especially in zebrafish (Rocha et al., 2017; Gallud et al., 2020). The zebrafish (*Danio rerio*) is an established bioindicator to study nanoparticle toxicity (Zhu et al., 2007; Martinez et al., 2019; Mishra et al., 2021). The specie is small in size, has good reproducibility and has a fast embryonic development. The embryo is transparent and thus the development of organs can be visualized during development (Wang et al., 2012; Paatero et al., 2017). Different parameters can be assessed, such as hatching, organ development, respiration, behaviour, immunotoxicity, and genotoxicity, in addition to reproductive toxicity and mortality.

Accordingly, the present study aimed to determine the embryotoxicity induced by three CdTe QDs functionalized with carboxylate (COOH), ammonia (NH_3_), and polyethylene glycol (PEG) using zebrafish embryo test (ZET) as a model. The authors conducted a sequence of assessments for embryonic developmental toxicity including embryonic mortality, hatching rate, malformation percentage, respiration rates and Cd/Te uptake from QDs exposure. Combining embryonic toxicity, metal body burden and respiration rates as indicators of evaluating QDs toxicity will give a clearer indication of the toxicity (nano-specific or metal-based) which is important for safety by design for biomedical application.

## 2 Materials and methods

### 2.1 Chemicals

Quantum dot nanopowders with 3–5 nm primary particle size were produced by PlasmaChem GmbH (Lot# YF140402) and supplied by the Nanosolutions EU FP7 project (Project ID: 309329). The stock suspensions (100 mg/L) were freshly prepared by directly dispersing the three types of QDs (5 mg) in 50 mL of ultrapure water individually (18.2 MΩ/cm, Millipore, Billerica, MA). The 50 mL nano stock solutions were sonicated at 60°C in a bath ultra-sonicator (Model E-UC6-HD-D, Scientech, India) for 1 h at 20 Hz prior to dilution in E3 media for exposure and characterization.

### 2.2 Preparation and characterization of quantum dots

Characterization was done in two batches. Firstly, the 100 mg/L NM stocks in ultrapure were characterised by the University of Plymouth as part of the Nanosolutions project. Secondly, QDs in E3 media at concentrations used for this study was characterized at the North-West University.

Characterization of 100 mg/L stock CdTe QDs data were obtained from the Nanosolutions project data sheets since they were the same batches that were used in this study (Nanosolutions., 2013; Vassallo et al., 2018; Tatsi et al., 2020). Briefly the hydrodynamic size distribution of stock solutions was determined by Nanoparticle Tracking Analysis (NTA) using Nanosight LM 10 (Nanosight, Salisbury, United Kingdom, laser output set at 30 mW at 640 nm) and methods described by Besinis et al. (2014). Material dissolution was measured using dialysis bags containing 700 μL of the appropriate stock solution. Briefly, 8 mL of a sonicated stock of each material was added to a dialysis tube (product code: D9777, cellulose membrane with molecular weight cut off at 12,000 Da, Sigma-Aldrich Ltd., Dorset, United Kingdom) which was then secured on both ends using cable ties to prevent leakage. Each bag was then placed in a beaker containing 492 mL of ultrapure water. Samples (15 mL) of the external water from each beaker was taken after 24 h. Each material was analysed in duplicate on a shaker (IKA Labortechnik, KS250 basic). Samples of 15 mL where acidified with 300 μL of 65% nitric acid. Metal concentrations were measured by inductively coupled plasma optical emission spectrophotometry (ICP-OES, ICAP 7400) and inductively coupled plasma optical mass spectrophotometry (ICP-MS, Thermoscientific, X Series 2).

The primary particle diameter of initial nanomaterial particles was determined using transmission electron microscopy (TEM) (FEI Tecnai G2). One drop of 100 mg/L solution of each individual nanomaterial was added to a carbon grid separately, allowed to settle and dried, thereafter it was examined at high resolution (200 kV).

For exposures, standard E3 media was used. The media was constituted by dissolving 34.8 g NaCl, 1.6 g KCl, 5.8 g CaCl_2_.2H_2_O, and 9.78 g MgCl_2_.6H_2_O in 2 L reverse osmosis water. The pH of approximately 7.6 remained stable at 7.65. To make up the desired exposure concentrations the relevant volumes of sonicated nanomaterial stock solutions (100 mg/L) were added to the exposure media. Six different concentrations were selected for each nanomaterial with 5 replicates of each. Nominal exposure concentrations were 0, 0.5, 2, 4, 6, and 20 mg/L QDs.

Further characterization of exposure solutions in E3 media was conducted at the North-West University. Hydrodynamic size distribution in E3 media was measured using Dynamic Light Scattering (DLS) and stability were examined with a zeta-sizer (Malvern, Zetasizer Nano-ZS). The total metal content of solutions was determined by acidifying solutions (10 μL 65% HNO_3_ per 10 mL) and measuring Cd and Te concentrations using standard ICP-MS techniques.

### 2.3 Zebrafish maintenance and embryo exposure

Long-fin wild-type adult *D. rerio* breeding stocks were kept in a ZebTEC recirculating aquarium system in an environmentally controlled room (28°C). Adult *D. rerio* breeding stocks were bred in Tecniplast iSpawn spawning chambers (Tecniplast, Italy) in a ratio of two males to three females. After approximately 2 h post fertilization (hpf) eggs were collected and placed in an incubator at 27°C for another 2 h (Martinez et al., 2019; Kumar et al., 2020). The *D. rerio* embryo acute toxicity test (OECD 236—OECD, 2013) was carried out using 4 hpf embryos. Viable fertilized eggs were sorted using a stereo microscope and separated from unhealthy eggs. Healthy fertilized eggs were then treated with QD-NH_3_, QD-PEG, and QD-COOH (0.5, 2, 4, 6, and 20 mg/L) respectively. Test concentrations of each material were prepared by adding the required amount of the stock solution to each well in a 6-well plate and filling it to 5 mL with E3 media. Negative controls consisted of E3 media with no material added. Five viable eggs were then transferred to each well, thus for each treatment consisted of four replicates of five eggs (*n* = 20). The six-well plates were sealed with self-adhesive, oxygen-permeable sealing film (BRAND^®^, Sigma Aldrich, Merck, Darmstadt, Germany) to prevent evaporation from taking place. Unless otherwise stated, data for the QDs are shown as a nominal mass concentration of QD per unit volume and not normalized to the concentration of metal in the particle. This is because the dissolution rates of QDs were very low and therefore the particles were the predominant form in which the metal occurred in the media throughout the exposures. The plates were then incubated for 96 h at 27°C at a 12:12 dark-light cycle. Mortality, hatching rate and malformations were noted every 24 h. Observations performed on each tested embryo included: Coagulation of embryos, lack of somite formation, non-detachment of the tail, spinal deformation, pericardial edema and lack of heartbeat.

### 2.4 Embryo respiration

Respiration was measured at 24 h and 48 h by removing embryos from exposure media, washing them twice with clean E3 media and placing 5 embryos per replicate in each well of a 12-well Loligo^®^ microplate system containing 80 µL of aerated E3 media. Three replicates were assessed at each concentration (*n* = 3). Measurements were taken every 5 s for at least 30 min, where after embryos were carefully removed from the wells and returned to the exposure media and incubated to continue with the test. Data are expressed as mg oxygen per embryo per minute.

### 2.5 Metal uptake in larvae

A parallel exposure was carried out concomitantly in E3 media to obtain enough biomass for metal analysis. Fertilised eggs were exposed in groups of 20 in 30 mL of exposure media. After 96 h, larvae were collected and larvae that were not hatched yet were manually dechorionated using a pair of forceps to eliminate possible QDs adhering to the chorion surface. Larvae were humanely killed in ice water, washed twice in ultrapure water and 10 individuals were pooled to represent a replicate sample. A minimum of three replicates per treatment was immediately frozen at −20°C until analyses.

Thawed samples were weighed and digested for 24 h in a Teflon heatblock with 1 mL nitric acid (65%, suprapure, Merck) at 65°C. Metal concentrations were measured using standard ICP-MS. Untreated organisms were used as negative controls and the natural background concentrations were subtracted from the concentrations measured in the exposed organisms. Concentrations are expressed as the total metal concentration on a wet weight basis. Analytical quality control was assured using certified reference materials (DORM-4 fish tissue) and is presented with instrumental detection limits in Table 1.

**TABLE 1:** Recovery rates (%) and detection limits of cadmium and tellurium from larval tissue and E3 media samples.

Metal ion from QD treatment	Recovery rates (%) Dorm-4 certified reference material	Detection limits in larvae tissue (μg/g dw)	Detection limits in E3 media (μg/L)
Cd	99 ± 5	0.30	0.11
Te	Not available	2.1	1.38

### 2.6 Statistical analysis

The dose-response function, namely, effect concentration where 50% of the population died (LC_50_ values) was calculated using non-linear regression analysis in ToxRat^®^ software. Effect data are expressed as mean ± standard error. Significance between controls and treatments and functionalised groups were determined by using One-way analysis of variance (ANOVA) and Tukey’s *post hoc* test. All data were tested for normality using the Shapiro-Wilk test. Data that did not meet the normality assumption were log-transformed prior to analyses. Differences were considered significant at *p* < 0.05.

## 3 Results

### 3.1 Characterization

The primary particle size of all three QDs was 3–5 nm as described by the manufacturers. Nanoparticle tracking analysis performed at the University of Plymouth (as part of the Nanosolutions project) on 100 mg/L stock solutions indicated that QD-PEG had the largest hydrodynamic size distribution in ultra-pure water (Table 3).

Dynamic light scattering analysis of exposure concentration in E3 media conducted at the North-West University indicated that QD-COOH produced the largest hydrodynamic size distribution in E3 media and a decline in hydrodynamic size distribution as concentrations increased could be seen in all three materials. There was also a time-dependent agglomeration of particles (Table 2). Large agglomerates were observed settling out in exposure vessels at the 20 mg/L concentration. Hydrodynamic particle size was smaller in ultrapure water compared to concentrations made in E3 media (Tables 2, 3). The zeta potential of QDs were measured in E3 media to provide quantitative information on the stability of QDs in this study. Overall no significant effects on zeta potential were observed. Dissolution was also measured as part of the Nanosolutions project, and it was established that all three groups had less than 4% metal dissolution in ultra-pure water, only the QD-NH_3_ group had a larger dissolution of Te at 23.69% (Table 3). Based on the total metal content of each concentration of QDs the Cd concentration was between 3.8 and 5.5-fold higher than the Te concentration in QD-COOH and QD-PEG but between 28 and 55-fold higher in QD-NH_3_. The QD-COOH group has the highest overall metal content and the QD-NH_3_ group the lowest. There was also an increase in total metal content as exposure concentrations increased.

**TABLE 2:** Hydrodynamic size (nm) distribution, zeta potential (mV) and metal concentrations (mg/L) of CdTe QDs in E3 media (±SD) measured at the North-West University.

			Exposure concentration (mg/L)				
Material	Endpoint	Time point (h)	0.5	2	4	6	20
QD-COOH	Hydrodynamic size (nm)	0	1,068 ± 345	704 ± 148	699 ± 79	649 ± 92	156 ± 31
		48	710 ± 91	1,212 ± 15	779 ± 49	782 ± 82	226 ± 36
	Zeta potential (mV)	0	−22 ± 3	−18 ± 13	−23 ± 1	−23 ± 0.6	−17 ± 10
		48	−24 ± 1	−13 ± 9	−22 ± 2	−23 ± 2	−19 ± 5
	Cadmium concentration (mg/L)	0	0.19 ± 0.03	0.9 ± 0.1	1.6 ± 0.05	2.5 ± 0.06	8.1 ± 0.3
	Tellurium concentration (mg/L)	0	0.034 ± 0.005	0.2 ± 0.03	0.3 ± 0.01	0.56 ± 0.02	1.6 ± 0.07
QD-PEG	Hydrodynamic size (nm)	0	697 ± 38	801 ± 43	682 ± 22	459 ± 21	93 ± 14
		48	1,003 ± 23	1,026 ± 156	924 ± 81	630 ± 69	163 ± 16
	Zeta potential (mV)	0	−22 ± 4	−15 ± 4	−15 ± 10	−14 ± 2	−9 ± 5
		48	−22 ± 1	−22 ± 1	−22 ± 1	−22 ± 2	−14 ± 7
	Cadmium concentration (mg/L)	0	0.2 ± 0.03	0.8 ± 0.05	1.6 ± 0.1	1.7 ± 0.5	6.9 ± 0.2
	Tellurium concentration (mg/L)	0	0.05 ± 0.006	0.15 ± 0.01	0.36 ± 0.02	0.44 ± 0.2	1.7 ± 0.05
QD-NH_3_	Hydrodynamic size (nm)^a^	0	880 ± 91	643 ± 12	489 ± 46	436 ± 17	84 ± 21
		48	769 ± 54	697 ± 50	690 ± 69	492 ± 12	120 ± 25
	Zeta potential (mV)	0	−22 ± 4	−21 ± 3	−21 ± 0.7	−7 ± 10	−7 ± 6
		48	−22 ± 2	−23 ± 0.5	−17 ± 11	−23 ± 0.4	−22 ± 2
	Cadmium concentration (mg/L)	0	0.04 ± 0.002	0.15 ± 0.04	0.33 ± 0.03	0.46 ± 0.02	1.6 ± 0.05
	Tellurium concentration (mg/L)	0	0.002 ± 0.0002	0.005 ± 0.001	0.006 ± 0.0002	0.01 ± 0.003	0.056 ± 0.005

^a^ Data refer to aggregate hydrodynamic diameters from particle size distribution measurements made by DLS, at the North-West University.

**TABLE 3:** Hydrodynamic size (nm) distribution and metal dissolution (mg/L) of 100 mg/L CdTe QDs stock solutions after 24 h in ultrapure water as analysed by Plymouth University for the Nanosolutions project (±SD).

Material		QD-COOH	QD-PEG	QD-NH_3_
Mean hydrodynamic size (nm)^a^		84 ± 58	159 ± 72	75 ± 50
Dissolution (%)^b^	Cd	0.74	2.43	2.10
	Te	0.39	3.23	23.69

^a^ Data refer to aggregate hydrodynamic diameters from particle size distribution measurements made by NTA, at Plymouth University.

^b^ The values have been calculated taking into account the degree of functionalization or impurities content as reported by Nanosolutions.

### 3.2 Metal uptake

**FIGURE 1:**
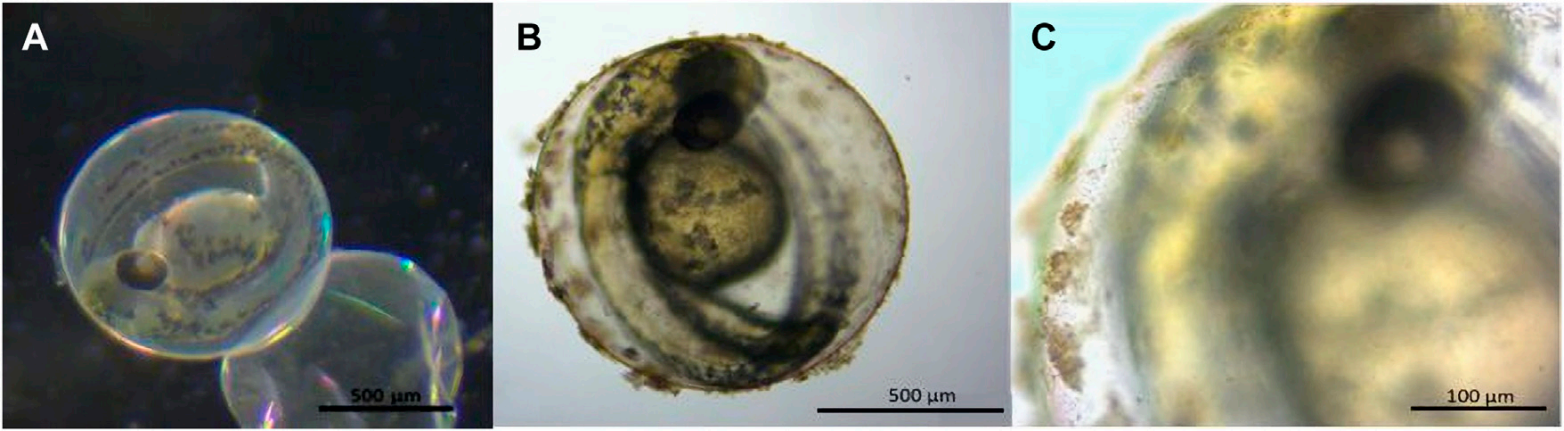
Stereo microscope images of 48 hpf *Danio rerio* embryos indicating no particle agglomerates on the chorion of the control **(A)** and QD agglomerates covering the outside of the chorion **(B, C)** during exposure to QD-NH_3_ 20 mg/L.

Particle agglomerates adhered to the surface of the embryo chorion in only the QD-NH_3_ group (Figure 1). Thus, for metal uptake, only hatched larvae after 96 h of exposure were used to avoid any metal from the chorion surface being included in uptake results.

**FIGURE 2:**
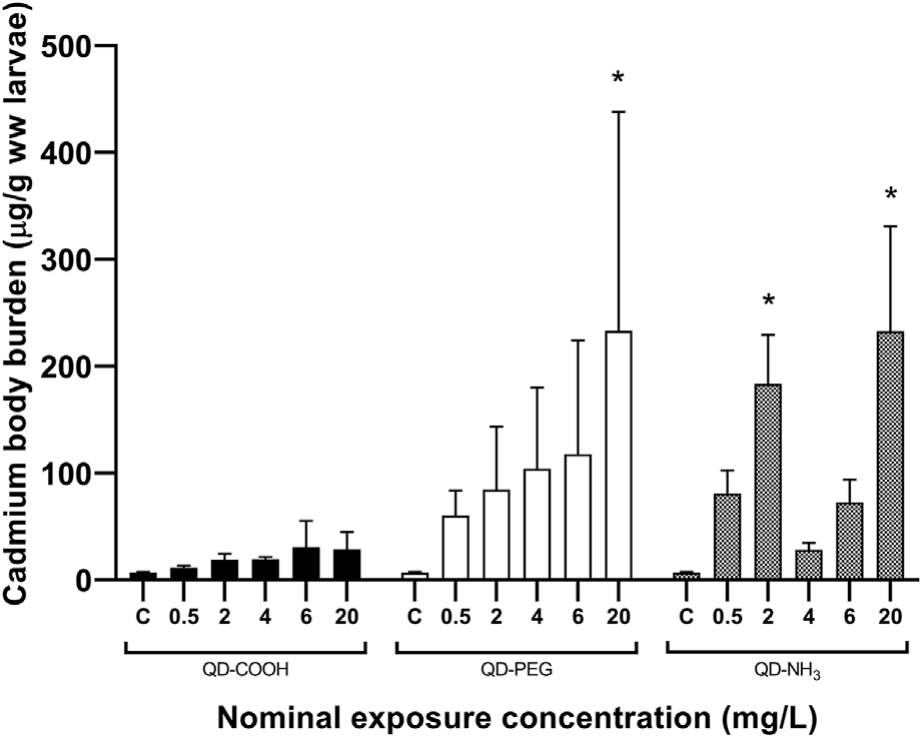
Total Cd concentrations (mean ± standard error expressed as µg/g ww larvae) in *Danio rerio* larvae after exposure to increasing concentrations of QD-COOH, QD-PEG and QD-NH_3_. Asterisk (*) indicates significance from control (*p* < 0.05, T-tests).

Body burden of Cd after 96 h of exposure showed no significant uptake of Cd from the QD-COOH group and the QD-PEG group showed a concentration dependant increase in Cd uptake with the only significant increase compared to control was seen at the highest exposure concentration (20 mg/L) (Figure 2). The QD-NH_3_ group showed no dose-dependent increase in Cd uptake. The only two exposure concentrations that showed significant uptake was 2 mg/L and 20 mg/L.

**FIGURE 3:**
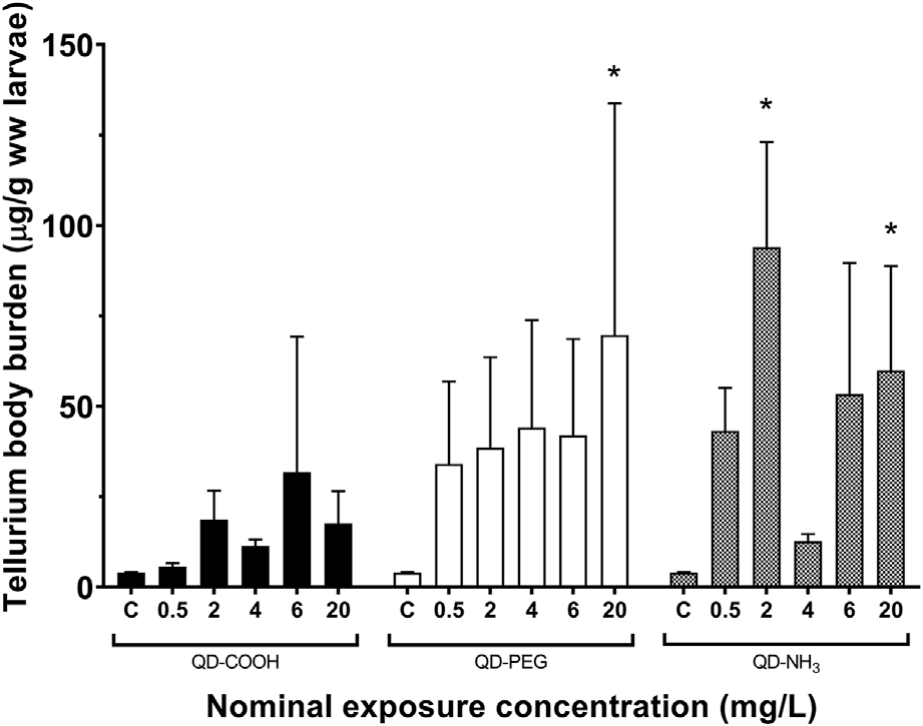
Total Te concentrations (mean ± standard error expressed as µg/g ww larvae) in *Danio rerio* larvae after exposure to increasing concentrations of QD-COOH, QD-PEG and QD-NH_3_. Single asterisk (*) indicates significance from control (*p* < 0.05, *T*-tests).

Similar uptake patterns were seen for Te but at lower concentrations (Figure 3). A significant uptake of 70 μg/g was found in the 20 mg/L exposure concentration of the QD-COOH group and the QD-NH_3_ group caused uptake of 94 μg/g and 60 μg/g in the 2 and 20 mg/L exposure concentrations.

### 3.3 Respiration

**FIGURE 4:**
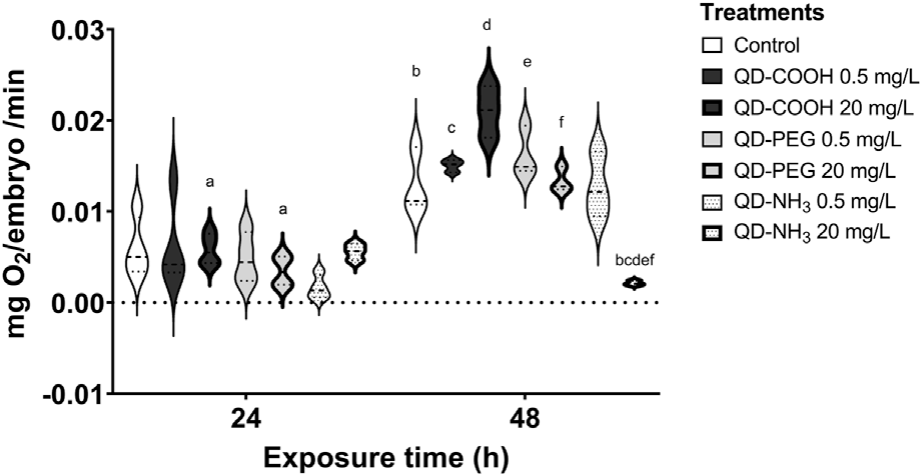
Respiration of *Danio rerio* embryos 24 hpf and 48 hpf. The graph represents the replicates and mean of each treatment. The same letters in superscript denotes a significant difference between treatments (*p* < 0.05, *T*-tests).

After 24 h there was no significant effect on the respiration of embryos (Figure 4). However, after 48 h the highest concentration of the QD-NH_3_ group caused a significant decrease in respiration compared to the control. The highest QD-COOH exposure group concentration (20 mg/L) resulted in a significant increase in respiration compared to the control.

### 3.4 Developmental toxicity

**FIGURE 5:**
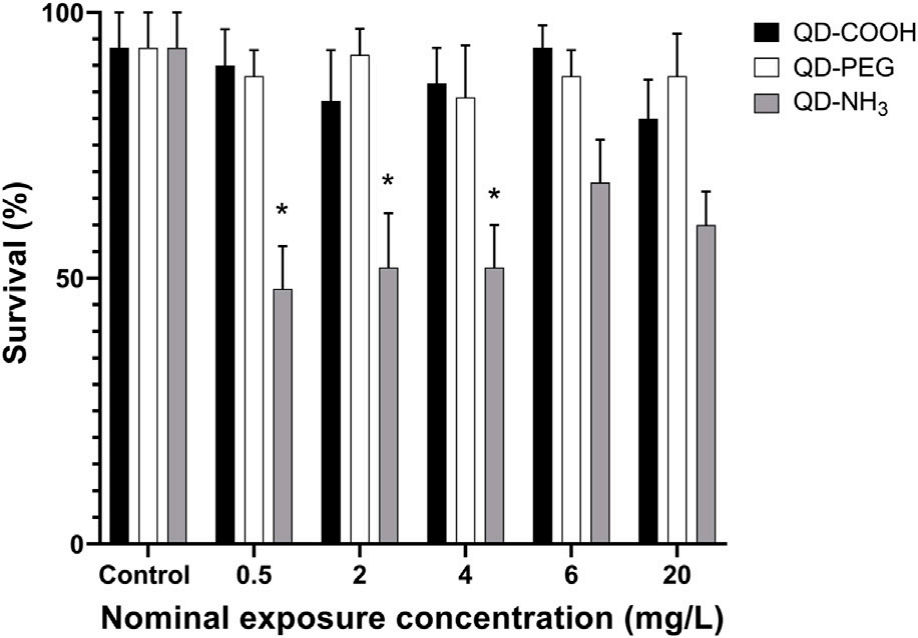
Survival of *Danio rerio* larvae after 96 h of exposure to QD-COOH, QD-PEG and QD-NH_3_. Error bars represent standard deviation. Single asterisk (*) indicates differences from control (*p* < 0.05).

Exposure to QD-COOH and QD-PEG groups did not produce any significant decrease in embryo survival (Figure 5). It was therefore not possible to calculate LC_50_ values for these groups, however the LOEC was calculated as ≥ 20 mg/L. Exposure to the QD-NH_3_ group caused significant mortality (i.e., > 50%) at the low concentrations of 0.5, 2, and 4 mg/L, while no mortality was observed at the higher exposure concentrations.

**FIGURE 6:**
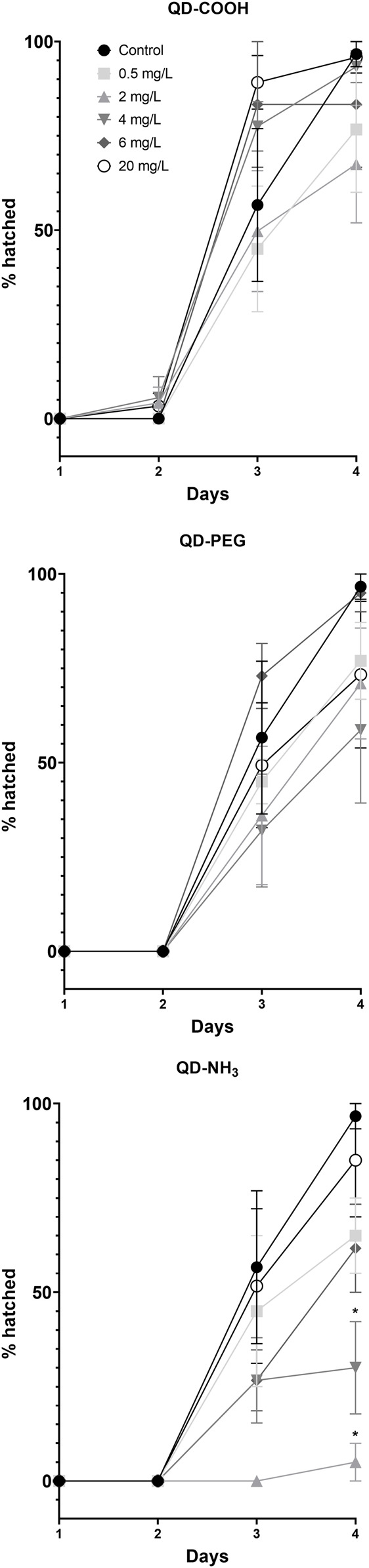
Percentage hatched *Danio rerio* larvae at different time points during the exposure to QD-COOH, QD-PEG and QD-NH_3_. Error bars represent standard deviation. Single asterisk (*) differences from control (*p* < 0.05, *T*-tests).

Two low concentrations (2 and 4 mg/L) of the QD-NH_3_ group caused a significant (*p* < 0.05) delay in the hatching of surviving embryos (Figure 6). No significant effect on hatching success during the exposure period was observed at the other exposure concentrations.

**FIGURE 7:**
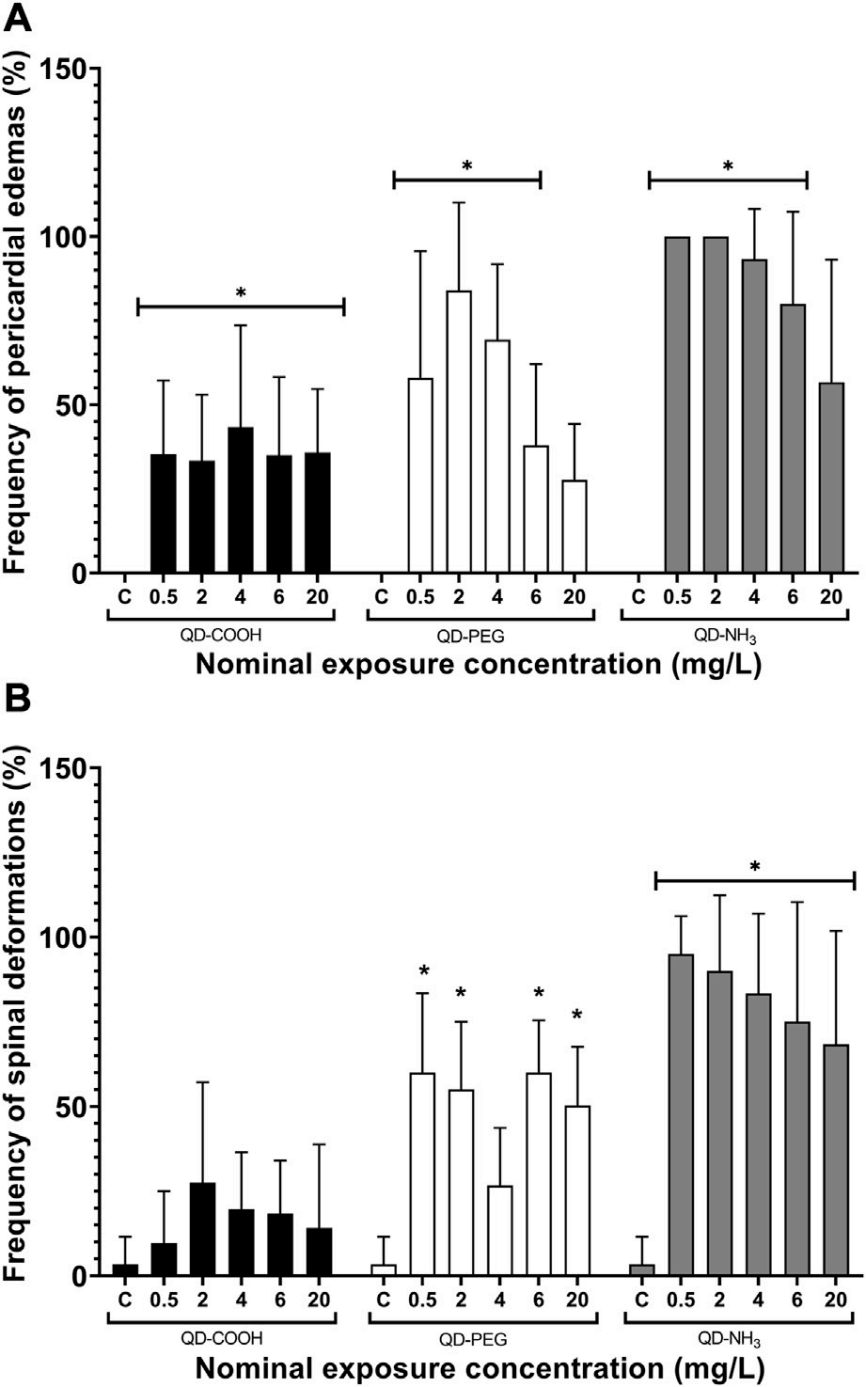
Spinal deformations **(A)** and pericardial edemas **(B)** of *Danio rerio* embryos exposed to QD-COOH, QD-PEG, and QD-NH_3_ expressed as a percentage of surviving embryos. Error bars represent standard deviation. Single asterisk (*) denotes significant differences from control (*p* < 0.05, *T*-tests).

Exposure to all concentrations, except for the highest exposure (20 mg/L), of QD-PEG and QD-NH_3_ resulted in significant increases in pericardial edemas (Figure 7A). All concentrations of the QD-COOH group also resulted in significant pericardial edemas (Figure 7A). No significant occurrence of spinal deformations was observed in embryos exposed to QD-COOH (Figure 7B). The QD-PEG group showed significant spinal deformations compared to the control at all exposure concentrations except he 4 mg/L exposure. The QD-NH_3_ group also showed a significant occurrence of spinal deformations at all concentrations, with the lowest concentration (0.5 mg/L) having the highest effect.

## 4 Discussion

Nanomaterials have many beneficial properties and as their daily use is increasing it is inevitable that this will lead to the exposure of humans and other organisms to these materials (Ghobadian et al., 2015). The size and functionalization of these materials influence their toxicity and thus it is important to investigate and compare the toxicity of different functionalized QDs with the same primary particle size. In this study, we showed that there are clear differences of particle behaviour in exposure media influencing toxicity, metal uptake and respiration effects to the zebrafish embryo’s during exposure. Overall, the QD-NH_3_ group resulted in the most significant negative effects to the developing embryos causing severe malformations, hatching delay and mortality at low concentrations, and respiration inhibition at the highest concentration (20 mg/L). This contrasts with findings by Zheng et al. (2021) that showed exposure to CdSe/ZnS QDs with COOH functionalization was more toxic to developing zebrafish than NH_3_ functionalized material. The QD-PEG group caused significant edema and spinal deformations in a bimodal pattern, caused no respiration effects and no significant mortality, while the QD-COOH group showed the least toxic effects with only significant cardiac edema and increased respiration rate the lowest exposure concentration.

### 4.1 Dissolution and colloidal activity of particles

The colloidal activity of these particles can be described by the DLVO theory. This suggests that both van der Waals forces and electrostatic forces cause aggregation of particles in a suspension (Adair et al., 2001). At higher concentrations van der Waals forces between the large number of particles cause higher aggregation of nanoparticles. At a certain point the aggregates and agglomerates become too large to remain in suspension causing them to settle out. This makes them less bioavailable and influences toxicity. In the present study it was seen that QD-COOH caused the largest aggregates in the exposure media. This can be attributed to Mg and Ca ions in the media that reduce the electrostatic double layer initiating binding with the negatively charged COOH chain on the nanomaterial (Deerfield et al., 1989). However, these interactions are dynamic and size, temperature, time and concentration dependent. Thus, bioavailability and toxicity and change over time and concentration, leading to the bimodal and effects at lower concentrations seen in the present study.

Dissolution is a fundamental parameter to be considered when nanomaterial toxicity is assessed, as it can lead to highly toxic ions interacting with target sites (Zolotarev et al., 2012). The QDs used in this study have a very low dissolution potential (<4%) in ultra-pure water and decreases in solutions with higher pH such as E3 media that was used in this study (Boyle et al., 2020). Thus, the effects of dissolution metal ions from these materials were not considered to be significant. The highest dissolution of Te was from the QD-NH_3_ group (i.e., 23%) meaning that there would only be 13 μg/L Te ions in the highest exposure concentration of 20 mg/L. This is less than 10% of the NOEC derived for Te in algae and freshwater fish (ECHA, 2022). Total body metal concentrations for both Cd and Te increased in a dose-dependent manner only in the QD-PEG group. There were however no clear dose-response relationships between the body burdens and the effects observed, suggesting mechanisms other than dissolved metal content and total internal exposure (as reflected in the body burdens).

However, it may be possible that despite the insignificant dissolution of metals in exposure media, *in vivo* QD degradation may expose embryos to Cd and Te (Gao et al., 2019). This would be expected to produce endpoints of Cd toxicity in zebrafish embryos exposed to sublethal QD concentrations. King-Heiden et al. (2009) found that CdSe QDs with different chain lengths did degrade internally into Cd^2+^ ions. The only toxicity parameter that can be linked to total metal uptake in our study was hatching success. A study by Zolotarev et al. (2012) also found hatching success impacted by Cd^2+^ ions dissociating from Cdse/Cds/Zns/S,S-Dihydrolipoic acid/Polyacrylic acid QDs, but also found edemas and spinal deformations associated with Cd^2+^ ions. Boyle et al. (2020) and Vicario-Parés et al. (2014) suggest that metal ions from nanomaterials bind to the hatching enzyme sites resulting in delayed or non-hatching of embryos. Delayed hatching in the present study (in the case of QD-NH_3_ exposures) also lead to decreased interaction of the larvae with the media thereby decreased uptake of nanomaterials due to the prolonged protection of the chorion; this was seen for the 4 mg/L concentration but not for the 2 mg/L concentration in the present study.

### 4.2 Embryo developmental toxicity

Hatching success has been linked to increased occurrence of developmental defects following exposure to metal nanomaterials (King-Heiden et al., 2009; Asharani et al., 2011; Zhao et al., 2013; Ghobadian et al., 2015; Zheng et al., 2021). In this study malformations were more prevalent in the QD-NH_3_ group at the lower exposure concentrations. This contrasts with studies by Duan et al. (2013), that found a very strong dose-response relationship between exposure concentrations and developmental toxicity. These contrasting findings are probably related to the difference in materials with Duan et al. (2013) using water-soluble CdTe QDs as opposed to the three functionalised groups used in this study. Pericardial edemas were the most common malformation observed, consistent with previous results (Zhang et al., 2012a; Xu et al., 2012; Duan et al., 2013; Jin et al., 2017). Cellular studies have also shown that NH_3_ functionalized graphene oxide nanoparticles were more cytotoxic than pristine particles and mammalian studies exploring PEG functionalized gold nanoparticles found that these PEGylated particles were toxic to rats when injected intravenously (Cho et al., 2009; Keremidarska-Markova et al., 2018). Tang et al. (2013) reported that QDs enter cells through endocytosis and cause the formation of reactive oxygen species (ROS), which result in ROS-mediated genotoxicity leading to malformations. Studies have also concluded that QDs and Cd ions cause pericardial edemas and in turn, impaired cardiac function via bradycardia (Zhu et al., 2009; Duan et al., 2013; Krzykwa et al., 2019; Zheng et al., 2021).

Since the cardiac function influences the respiration rate, we established whether exposure to the three different functionalised QDs influenced the oxygen consumption of zebrafish embryos. Data showed that the embryo oxygen consumption displayed a different trend from the other effects recorded. The QD-NH_3_ group had the greatest effects but only at the highest exposure concentration (20 mg/L). Large agglomerates formed and adhered to the chorion of the embryos. This was also reported in other studies (Sun et al., 2013; Petushkova et al., 2015; Zheng et al., 2021). The agglomerations were the result of increased ZP at the highest exposure concentration and settling out of suspension. Lower dispersions ZP values will lead to aggregation, coagulation, or flocculation due to van der Waals interparticle attraction (Joseph and Singhvi, 2019; Shnoudeh et al., 2019), as is the case in the present study where all ZP values were in the non-stable range.

The chorion consists of two membranes, namely, the chorion membrane and the vitelline, it also has pores running through it to assist in gas exchange, as gills only develop after 2 weeks (Pelster and Bagatto, 2010). The pore canals are between 0.5 and 0.7 µm in diameter, this means that small particles can permeate these canals and enter the larvae. However, larger agglomerates can also block these canals causing hypoxic conditions for the embryo (Lee et al., 2007; Vicario-Parés et al., 2014; Samaee et al., 2015; Lacave et al., 2016). Even though embryos were washed in clean media before respiration measurements were taken there still could have been residual agglomerates blocking pore canals and impairing respiration in the 20 mg/L QD-NH_3_ treatment embryos. Increased oxygen consumption is most likely due to stress, increased activity or increased metabolic activity seen in the 20 mg/L QD-COOH group, as no agglomerates on the embryo surface were observed in this treatment. Increased oxygen requiring activities were observed as severe hyperactivity in larvae exposed to low doses (2.5 nM) of uncoated CdTe QDs (Duan et al., 2013), as well as anxiety-related swimming behaviour changes after exposure to CdSe QDs (Zonouzi-Marand et al., 2022).

By comparing responses of zebrafish embryos following exposure to different functional groups QDs, it was evident that developmental and respiratory effects could not solely be attributed to metal ion toxicity. This supports findings by Tang et al. (2013) that reported effects of free Cd could not adequately explain QD toxicity in zebrafish embryos. Endpoints assessed in the present study also indicated a clear difference in the severity of responses between functionalized groups. Bimodal responses in metal uptake and malformations were observed for QD-PEG and QD-COOH treatments. This is most likely attributed to smaller NMs crossing the chorion membrane and eliciting direct toxicological responses, whereas larger agglomerates at higher exposure concentrations blocking the membrane pores and eliciting indirect toxicity. Bioaccumulation and elimination rates are important parameters in QD toxicokinetics and merit examination (King-Heiden et al., 2009). The QD-COOH and QD-PEG groups may be less bioavailable compared to the QD-NH_3_ group. Work by King-Heiden et al. (2009) found that QD-PEG with different chain lengths did exhibit toxicity towards zebrafish embryos but these effects were less severe than non-functionalized QDs. Several studies suggest that the terminal functional group of QDs and surface chemistry influences tissue distribution (Hardman, 2006). Thus, it is likely that surface chemistry, surface charge, and particle size influenced particle bioavailability and mode of toxicity, accounting for the differences observed in this study.

## 5 Conclusion

In summary, this study demonstrates that CdTe QDs cause developmental embryonic toxicity, resulting in persistent effects on larval development and respiration. We also established that different functionalized CdTe QDs materials tended to agglomerate and react differently in exposure media, which in turn influenced the toxicity. The QD-NH_3_-were the most toxic of the three tested QDs in this study. It was also seen that lower exposure concentrations were more toxic to survival and spinal deformations compared to high exposure concentrations, and no dose dependent effects were observed for all three materials in this study regardless of metal uptake. This was attributed to changes in the agglomerate sizes and colloidal behaviour of the particles in the media. This was evident in larger agglomerates forming at the high exposure concentrations, which resulted in decreased respiration rates, such as for the QD-NH_3_ group. It is thus concluded that three differently functionalized quantum dots with the same primary particle size can have different toxicities due to the particles characteristics, and that the toxicity observed is not solely due to metal body burden but rather a combination of particle specific effects due to aggregation, such as respiration impairment and metal uptake at lower concentrations. This highlights the need for further investigation.

## Data Availability

The raw data supporting the conclusion of this article will be made available by the authors, without undue reservation.
